# Robust Iris-Localization Algorithm in Non-Cooperative Environments Based on the Improved YOLO v4 Model

**DOI:** 10.3390/s22249913

**Published:** 2022-12-16

**Authors:** Qi Xiong, Xinman Zhang, Xingzhu Wang, Naosheng Qiao, Jun Shen

**Affiliations:** 1 MOE Key Lab for Intelligent Networks and Network Security, School of Automation Science and Engineering, Faculty of Electronic and Information Engineering, Xi’an Jiaotong University, Xi’an 710049, China; 2 International Collage, Hunan University of Arts and Sciences, Changde 415000, China; 3 School of Computing and Information Technology, University of Wollongong, Wollongong, NSW 2522, Australia

**Keywords:** iris localization, iris recognition, radial gradient amplitude, YOLO, modified integro-differential operator, biometric, Daugman’s operator, MobileNet

## Abstract

Iris localization in non-cooperative environments is challenging and essential for accurate iris recognition. Motivated by the traditional iris-localization algorithm and the robustness of the YOLO model, we propose a novel iris-localization algorithm. First, we design a novel iris detector with a modified you only look once v4 (YOLO v4) model. We can approximate the position of the pupil center. Then, we use a modified integro-differential operator to precisely locate the iris inner and outer boundaries. Experiment results show that iris-detection accuracy can reach 99.83% with this modified YOLO v4 model, which is higher than that of a traditional YOLO v4 model. The accuracy in locating the inner and outer boundary of the iris without glasses can reach 97.72% at a short distance and 98.32% at a long distance. The locating accuracy with glasses can obtained at 93.91% and 84%, respectively. It is much higher than the traditional Daugman’s algorithm. Extensive experiments conducted on multiple datasets demonstrate the effectiveness and robustness of our method for iris localization in non-cooperative environments.

## 1. Introduction

Biometric identification has emerged as a critical method for ensuring information security [1]. It is a process that uses some inherent and some unique physiological or behavioral features of human beings to determine their identity. The face [2,3,4], fingerprint [5], palmprint [6,7], and iris [8,9,10] are all common biometrics.

**Figure 1:**
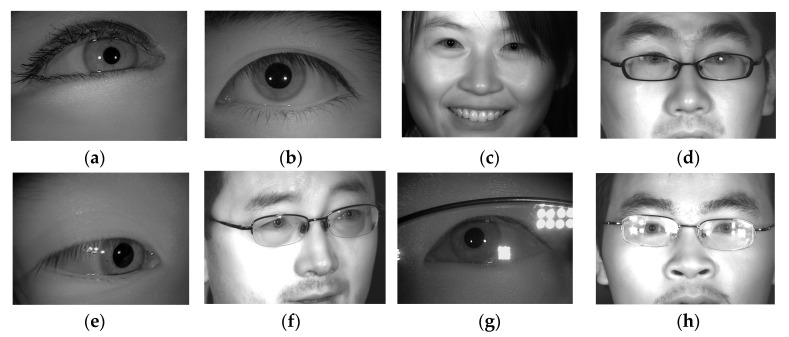
Some typical iris images obtained in non-cooperative environments: (**a**) iris obscured by eyelids; (**b**) iris interfered by eyelashes; (**c**) iris obstruction due to hair; (**d**) glasses obstructing the iris; (**e**,**f**) off-angle iris; (**g**,**h**) iris with specular reflection [22].

The iris is an area of the human eye that is approximately a circle between the sclera and the pupil. Unlike other biometrics, the iris has unique characteristics, such as a hidden location, being non-contact, and a rich texture. It is frequently used in identification and disease diagnoses [11,12]. Typically, iris segmentation and iris recognition are the two main tasks of the iris recognition system [13,14]. The purpose of iris segmentation is to distinguish iris and non-iris regions [15,16,17,18,19]. In addition, iris recognition needs to detect the pupil and iris circles, called iris location [19,20,21]. Its goal is to normalize the iris region into rectangles to prepare for iris recognition. Iris recognition and computer-aided eye disease diagnoses rely on an accurate iris location. The quality of the iris location directly affects the performance of these algorithms. However, iris-localization tasks are complicated due to non-cooperative environments, such as glasses reflections, an off-angle iris, long distances, occlusion by eyelashes or eyelids, being partially recorded, etc., as shown in Figure 1 [22]. As a result, developing a robust iris-localization algorithm is a challenging task with significant theoretical and application value, and it is rapidly becoming a hotspot in iris-recognition research [23,24,25,26]. Iris texture information will be lost if the location is incorrect, reducing the effectiveness of identification or disease diagnosis.

At present, there are mainly two types of iris-localization methods. One is the traditional algorithm based on machine learning. The other one is based on deep learning. The conventional way does not need to train many neural networks, which is easy to implement and very fast. The disadvantage is that it is susceptible to noise interference, has low accuracy, and has limited application scenarios. Deep-learning-based methods have a strong performance against noise interference and they have high precision. Deep learning models, however, need a long training period and lots of labeled data. The expense of labeling data severely limits their applicability to expand into new categories.

Motivated by the convenience of the traditional iris-localization algorithm and the accuracy of deep learning, this paper proposes an iris-localization algorithm based on a modified YOLO v4 network and a modified integro-differential operator. The following is a summary the main contributions:

(1) A modified YOLO v4 network is proposed to detect the iris region and locate the outer circle of the iris. We use MobileNetV2 as the backbone network in YOLO v4 for feature extraction. The modified YOLO v4 model is only 5.8 M in size, which is much smaller than the traditional YOLO v4-tiny model, which is 21.42 M in size. In addition, it also improves the mAP (mean average precision). It addresses the problem in which traditional localization algorithms are prone to noise interference and suffer from low accuracy.

(2) A modified integro-differential operator is proposed to precisely locate the inner and outer boundaries of the iris. The location effect of Daugman’s integro-differential operator is mostly promising. However, if the image is disturbed, iris localization is prone to failure. According to the principle of Daugman’s integro-differential operator, this paper proposes a modified integro-differential operator with better robustness. After removing some noise in the image, we can improve the accuracy in locating the inner and outer boundary with this modified operator. The experimental data show that the proposed localization algorithm can achieve high accuracy under non-cooperative environments. It has good robustness regardless of short-distance and long-distance irises.

(3) Strong scalability. The iris-localization method proposed in the paper combines the benefits of deep learning and machine learning. We can achieve accurate localization of the inner and outer circles of the iris without excessive labeling. For example, we only label 5% of the CASIA-Iris-Thousand dataset. The experiment shows that we can not only locate the labeled iris images, but also achieve high location accuracy with the remaining unlabeled images.

The remaining sections are structured as follows. In Section 2, we examine some of the literature regarding iris localization. Then, in Section 3, we go over the proposed methods in greater detail. The experimental findings in Section 4 show the effectiveness of the proposed method. Finally, we conclude in Section 5.

## 2. Related Works

Currently, there are primarily two categories of iris-localization techniques. One is the conventional machine-learning-based iris-localization algorithm. The alternative approach relies on deep learning.

### 2.1. Traditional Iris-Localization Algorithm

Traditional iris-localization algorithms are mainly divided into three categories. The first method is based on differential and integral operators. In this view, the iris is regarded as an approximate circle area. The iris location can be simplified by calculating the center and radius of the inner and outer boundaries. Daugman [27,28] used the integro-differential operator to search those parameters of the circle. Because this method needs to traverse and explore all parameter spaces, its computational complexity is relatively high. The second method is based on the Hough transform. Wildes [29] used the edge-detection operator to explore the edge points of the binary iris image, and then used the Hough transform to determine the parameters of the inner and outer boundaries. The third method is the gray difference method. This method fully uses the gray level changes of the iris image to locate the iris. Ma Li et al. [30] used gray value mutation to find three points that were not on the same line, and then combined them into a circle to determine the inner and outer boundaries of the iris.

### 2.2. Localization Algorithm Based on Deep Learning

In recent years, deep learning has rapidly developed in the field of target detection. There are currently two main approaches: one-stage detection and two-stage detection. The two stages are based on the concept of a target candidate box, generating a series of sample candidate boxes in advance, and then classifying samples through a convolutional neural network. The region-based CNN (R-CNN) is one of them. Feng X et al. [19] proposed an iris R-CNN. It can simultaneously complete the accurate segmentation and localization of the iris in a non-cooperative environment under visible light. Li Y H et al. [31] designed a fast R-CNN with only six layers to locate eyes. He used the bounding box found by Faster R-CNN to locate the pupil area using the Gaussian mixture model. Because R-CNN needs to select thousands of proposed areas from one picture, the speed is very slow. One-stage detection is based on regression, and there is no target candidate box. Image features extracted via the backbone network are returned to the object boundary box directly. It is faster than R-CNNs, such as YOLO models [32,33,34,35,36]. YOLO has attracted wide attention since its inception.

Many existing researchers have used the YOLO algorithm to locate the iris. Naranpanawa D et al. [37] proposed a light and simple object-detection model based on YOLO v3 to detect freckles in the iris. Evair Severo et al. [38] designed an iris target-detector based on YOLO. This target detector uses a small rectangular box which tightly encloses the iris region. However, this method cannot determine the position of the inner circle of the iris. Eduardo et al. [39] implemented a real-time iris-detection and segmentation framework in video based on Tiny-YOLO. The size of Tiny-YOLO is slightly larger so it is not suitable for installation in embedded systems.

In addition to the iris-localization algorithm based on YOLO, many deep neural networks that integrate the segmentation and location of the iris have emerged in recent years. Most of them are based on U-Net. Lian S et al. [40] introduced an attention mechanism to an original U-Net model to separate the iris and non-iris pixels. Wang C et.al [41] presented a multi-task U-Net called IrisParseNet. It can predict the iris mask, pupil mask, and iris outer boundary simultaneously. Interleaved Residual U-Net, proposed by Li Y H et al., can localize the outer and inner boundaries of the iris image [42]. Although the accuracy of the above methods is quite convincing, they are only used for segmenting and locating the iris from images of the eye region. They cannot help with extracting the eye region from long-distance iris images.

## 3. Methods

**Figure 2:**
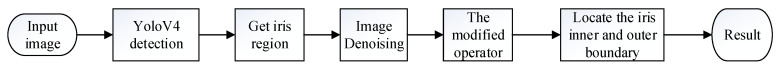
Flow chart of the localization algorithm.

We used MobileNetV2 to improve YOLO v4 and design an iris detector. The detector employs a small rectangular box that tightly surrounds the iris region. The iris region allows us to approximate the position of the pupil center. The improved integro-differential operator was used to precisely locate the inner and outer boundaries of the iris, greatly improving iris location robustness. Figure 2 depicts the flow chart of the localization algorithm.

### 3.1. Dataset

**Figure 3:**
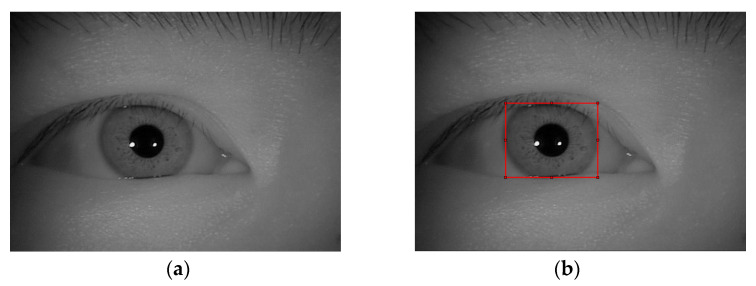

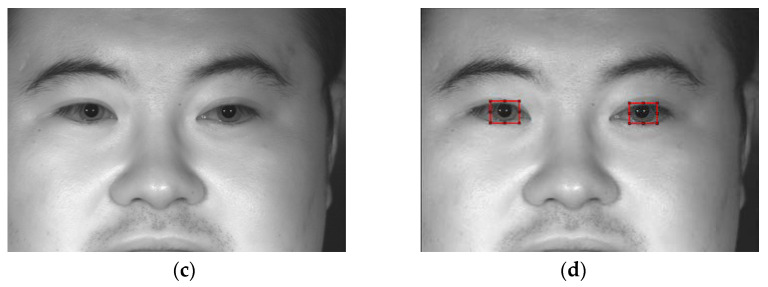
Sample image of the dataset used to train YOLO V4. (**a**) Example image of CASIA-Iris-Thousand; (**b**,**d**) Manual label of the outer boundary of the iris; (**c**) Example image of CASIA-Iris-distance [22].

When training the YOLO v4 network, we selected two datasets in this paper. One is CASIA-Iris-Thousand, and the other is CASIA-Iris-Distance [22]. The two datasets are briefly introduced below:

The dataset CASIA-Iris-Thousand includes 20,000 pictures of the iris from 1000 people. These images were collected at a short distance with an IKEMB-100 camera. Each image has 640 × 480 pixels. Changes in pupil size under different lighting conditions, as well as specular reflection, are the main causes of intra-class changes in CASIA-Iris-Thousand. Figure 3a shows a sample of the dataset.

The iris images in the CASIA-Iris-Distance dataset were captured by a high-resolution camera over a long distance. As a result, the image area of interest included both binocular irises and facial patterns. It contained 142 themes and 2567 pictures. Each image had 2352 × 1728 pixels. Figure 3c is a sample of the dataset. The imaging system of the high-resolution camera can actively search for patterns in the field of view, such as the iris and face, to identify users from a distance of about 3 m.

The number and size of the rectangular boxes manually labeled varied due to different datasets as shown in Figure 3b,d [22].

### 3.2. The Modified YOLO v4 Network

**Figure 4:**
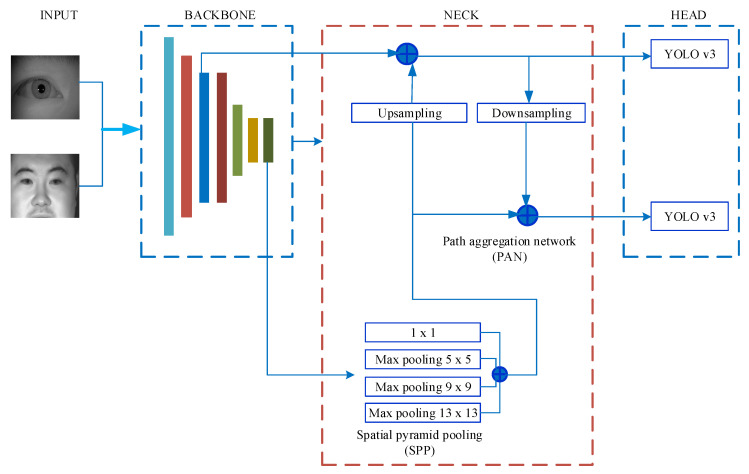
The overall network structure of modified YOLO v4 [44].

Three components make up the structure of the YOLO v4 network: the Backbone; the Neck; and the Head. CSPDarkNet-53 serves as the backbone of the traditional YOLO v4 network, which uses it to extract features from the input photos. This paper used the lightweight network MobileNetV2 [43] as the backbone network instead of CSPDarkNet-53. Its overall network structure is shown in Figure 4 [44].

#### 3.2.1. The Backbone

MobileNet was proposed by Google in 2017. MobileNetV2 further improves network performance by using a reverse residual structure [43]. Because MobileNetV2 contains seven bottleneck modules, we used seven blocks in the backbone shown in Figure 4. The backbone outputs the feature maps which are fused at the next part, namely the neck.

#### 3.2.2. The Neck

The head and the backbone are joined by the neck. A spatial pyramid pool (SPP) module and a path aggregation network (PAN) make up the neck. The head receives feature maps as input from the neck, which connects feature maps from various layers of the backbone. The SPP module uses kernels of size 1 × 1, 5 × 5, 9 × 9, and 13 × 13 for the max-pooling operation. The stride value was set to 1. The receptive field of the backbone features is expanded by concatenating the feature maps, which also improves the ability of the network to identify small objects.

#### 3.2.3. The Head

The head is responsible for receiving and processing a group of aggregation feature maps output by the PAN module. It predicts bounding boxes, classification scores, and objectivity scores. Three detecting heads are present in the head part of the traditional YOLO v4 network. Each detector head is a YOLO v3 network, and the respective output size is 19 × 19, 38 × 38 and 76 × 76.

Figure 3b,d shows that there were no more than two detection objects in a single image. Therefore, only two detection heads were required for the head. At the same time, because the size of the dataset was different, two feature maps with different sizes needed to be used for prediction. We extracted feature maps from the fourth and final bottleneck to predict small and large irises based on these specific situations. The network outputted feature maps of size 28 × 28 and 7 × 7.

### 3.3. Denoising an Iris Image

The rectangular area generated after iris detection can be regarded as the outer circle boundary of the iris. The center of the outer circle may not equal the actual pupil center, but it usually falls within the pupil. Removing noise interference without destroying the original image can provide a solid foundation for subsequent accurate localization.

It can be seen from Figure 3b,d that in the rectangular area, there were primarily two types of noise: reflective points and eyelashes. For the reflective points, we generated a mask based on those points. After we filled in regions specified by the mask using inward interpolation in the image, those reflective points were filtered. For the eyelash noise, it can be viewed as a dark detail in an image. We used morphological closure to fill the image and reduce the interference of the eyelashes.

### 3.4. Precise Localization of Iris Inner and Outer Boundaries Based on Improved Calculus Operator

#### 3.4.1. Daugman’s Integro-Differential Operator

The gray value of the iris image had noticeable changes at the inner and outer boundaries. Daugman proposed an integro-differential iris-localization algorithm based on this feature [26]. The mathematical expression is shown in Formula (1).

(1) $$\max_{\left(r,x_{0},y_{0}\right)}\left|G_{\sigma }\left(r\right)*\frac{\partial }{\partial r}\oint_{r,x_{0},y_{0}}\frac{I(x,y)}{2\pi r}ds\right|$$

where I(x,y) is the gray value of the point (x,y), $\oint_{r,x_{0},y_{0}}\frac{I(x,y)}{2\pi r}ds$ is the curve integral of the circle with center $\left(x_{0},y_{0}\right)$ and radius *r*. The integral path for the inner iris boundary is the entire circle. The outer boundary of the iris is easily interfered with by eyelashes and eyelids. The integration path for the outer iris boundary is the region with 90 degrees on the left and right sides of the iris. $G_{\sigma }\left(r\right)$ is a Gaussian function with standard deviation σ. * denotes convolution computation.

#### 3.4.2. The Modified Integro-Differential Operator

**Figure 5:**
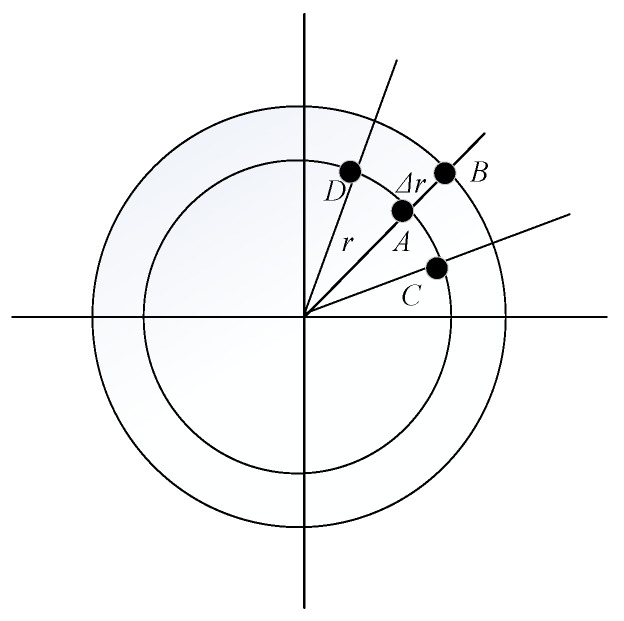
Schematic diagram of $g_{\theta ,r}$ and $C_{\theta ,r}$.

We can achieve good results by using Formula (1) to localize the inner and outer boundary of the iris with accurate pupil center positioning. If the pupil center positioning is skewed, the effect of iris localization is inferior to ideal. Based on the principle of Daugman’s integro-differential operator, we propose a modified operator with better robustness, as shown in Formula (2) [45].

(2) $$\max_{\left(x_{0},y_{0},r\right)}\sum_{\theta =1}^{n}\left(g_{\theta ,r}-C_{\theta ,r}\right)$$

where, the center and radius values of the search starting point are represented by $\left(x_{0},y_{0}\right)$ and radius *r*, respectively. *n* denotes the number of points taken uniformly around the circle, with $\left(x_{0},y_{0}\right)$ as the center and *r* as the radius. $g_{\theta ,r}$ represents the radial gradient of the $\theta^{\mathrm{th}}$ point on the circle of radius *r*. $C_{\theta ,r}$ is the compensation factor. $g_{\theta ,r}$ and $C_{\theta ,r}$ can be expressed by Formulas (3) and (4), respectively. Figure 5 depicts their schematic diagram.

(3) $$g_{\theta ,r}=I_{\theta ,r+\Delta r}-I_{\theta ,r}$$

(4) $$C_{\theta ,r}=\frac{1}{2}\left[\left|g_{\theta +1,r}-g_{\theta ,r}\right|+\left|g_{\theta -1,r}-g_{\theta ,r}\right|\right]$$

In Figure 5, point A represents the $\theta^{\mathrm{th}}$ point on the circle of radius *r*. Point B represents the $\theta^{\mathrm{th}}$ point on the circle of radius r+Δr. Points C and D represent the ${\left(\theta -1\right)}^{\mathrm{th}}$ point and the ${\left(\theta +1\right)}^{\mathrm{th}}$ points on the circle of radius *r*, respectively. The difference of gray value between point A and point B is $g_{\theta ,r}$.

To avoid error localization caused by the excessive radial gradient of an interference point on the circle, we introduced a compensation factor $C_{\theta ,r}$. If the difference of gray value between the two adjacent points on the circle was large, this indicated that these points were likely to be some interference points in the image.

#### 3.4.3. Localization of the Iris Inner Boundary

The center and radius parameters must be continuously varied in order to find the maximum value when applying Formula (2) to find the inner boundary of the iris. The initial value of the center $\left(x_{0},y_{0}\right)$ can be determined by the outer circle of the iris detected by YOLO v4. Because the center obtained by YOLO v4 is very close to the real center of the pupil, a smaller search field can be set to reduce the number of iterations and to speed up the localization. The search range of the inner circle radius can be set according to the prior conditions of the iris image. Because the inner circle of the iris is short, *n* can be set as 32. When Formula (2) returns the maximum value, we can obtain the center $\left(x_{p},y_{p}\right)$ and radius $r_{p}$ of the inner boundary of the iris.

#### 3.4.4. Localization of Iris Outer Boundary

The centers of the iris inner and outer boundaries are generally very close. When locating the outer circle, the search range of the circle center should be limited within a tiny neighborhood of the inner circle center. The radius search range can be based on the rectangular box obtained by YOLO v4. Its range is:

(5) $$1.2r_{p}<r_{1}<0.5\times \max[rows,cols]$$

where $r_{p}$ is the radius of the iris inner boundary obtained in Section 3.4.3.

$r_{1}$ is the search range of the radius of the outer boundary. rows,cols represents the length and width of the rectangular box obtained by YOLO v4 when the iris is detected. *n* can be set as 256. When Formula (2) returns the maximum value, we can obtain the center $\left(x_{i},y_{i}\right)$ and radius $r_{i}$ of the outer boundary.

## 4. Experimental Results and Analysis

### 4.1. Iris Images Pre-Processing

To verify the robustness of the method proposed in this paper, some iris images with poor quality, insufficient clarity, eyelash occlusion, and serious eyeglass reflection were not deliberately eliminated in the experiment. In the CASIA-Iris-Thousand dataset, we select 1000 images from the first 50 people. In the CASIA-Iris-Distance dataset, we selected 1000 images from the first 54 people. Image Labeler provided by Matlab 2022a was used to label those 2000 images. A total of 3000 rectangular boxes were labeled. Among those 2000 images, 80% were randomly selected as the training set and 20% were selected as the test set.

Because the input size of MobileNetV2 used in this paper was 224 × 224 × 3, prior to the experiment, the pre-processing process must uniformly convert the size of these 2000 images to 224 × 224 × 3. This is not only to meet the input requirements of MobileNetV2, but also to adapt to color iris images.

### 4.2. The Experimental Platform and the Evaluation Indicators

The experiment software and hardware platforms used were as follows: 64-bit Windows 10 operating system; Intel Core i7-8700 3.20 GHz dual-core CPU; 16 G running memory; and NVIDIA™ GeForce RTX 3060 GPU with 12 GB of memory. The software development environment was Matlab 2022a. The hyperparameters of the training model were set as follows: the training batch BatchSize = 4; the number of model training epoch = 5; the learning rate was 0.005; and the Adam algorithm was used for optimization calculation. All the experiments were run on one GPU card.

We used the average precision (AP) for comparisons in order to quantitatively evaluate the performance of different iris-detection algorithms. The AP was related to precision and recall, which can be formulated as [38]:

(6) Recall=TP/(TP+FN)

(7) Precision=TP/(TP+FP)

where the letters *TP*, *FP*, and *FN* stand for the numbers of true positives, false positives, and false negatives, respectively. *TP* (true positive) means that prediction is consistent with the label. *FP* (false positive) indicates that a negative case is predicted as a positive case, and *FN* (false negative) indicates that a positive case is predicted as a negative case. Usually, all iris region proposals with ≥0.5 IoU that overlap with a ground-truth box are considered *TP*, while others are considered *FP* [46]. The mean AP (mAP) function determines the average AP value across all object categories. The AP and mAP are quantitative indicators used in object detection. Generally, the higher the AP, the better the detection performance.

### 4.3. Comparison Experiment with Traditional YOLO v4

There are two types of backbone in the traditional YOLO v4 network: one is csp-darknet53-coco, and the other one is tiny-yolov4-coco. The COCO dataset was used to train these two networks. The size of the detection model with csp-darknet53-coco as the backbone is usually greater than 200 M. Because iris detection is mainly used in mobile phones or embedded devices, we need a smaller detection model. Although the precision of YOLO v4-darknet53 is very high, it is not suitable for embedded devices. Therefore, the comparison experiment here did not include YOLO v4-darknet53.

When the hyperparameters of model training are consistent, the experimental results show that the size of the YOLO v4-tiny model is 21.42 M, while the YOLO v4-MobileNetV2 model proposed in the paper is only 5.8 M, which is much smaller than the traditional YOLO v4-tiny model.

**Figure 6:**
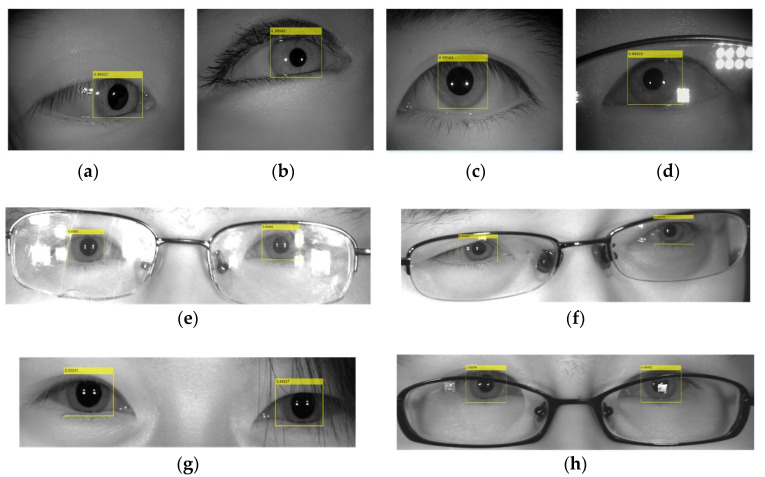
Some results of typical iris images obtained in non-cooperative environments. (**a**) Off-angle iris; (**b**) iris obscured by eyelids; (**c**) iris interfered with by eyelashes; (**d**,**e**) iris with specular reflection; (**f**) off-angle iris at a long distance; (**g**) iris obstruction due to hair; (**h**) glasses obstructing the iris [22].

Some results of iris localization under non-cooperative environments are shown in Figure 6. The yellow rectangle represents the iris-localization result. It can be seen that the iris-detection algorithm proposed in this paper has good anti-interference performance and strong robustness. We compared the mean average precision (mAP) of the iris under a different IoU in Table 1.

**Figure 7:**
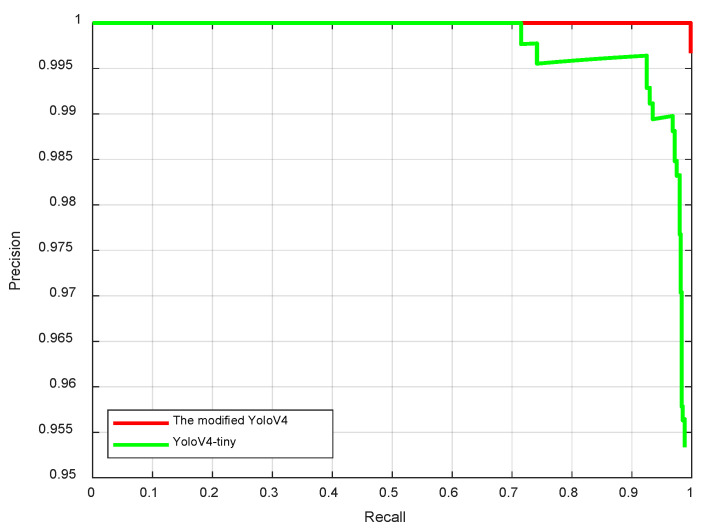
Precision recall curves of the two networks (IoU = 0.5).

From Table 1, we can see that when IoU was less than or equal to 0.7, the performance of the modified YOLO v4 model proposed in this paper was better than that of the traditional YOLO v4-tiny model. However, when the IoU was equal to 0.8, the mAP of the modified YOLO v4 model was significantly reduced. However, this had no effect on subsequent iris inner and outer circle localization. In our experiment, we set the IoU threshold to 0.5. In this case, the recall curves of the two networks are shown in Figure 7.

From Figure 7, we can see that the mAP of the modified YOLO v4 network was close to 100% when epoch = 5, and the performance was better than that of the traditional yolov4-tiny network.

### 4.4. Experiment with Inner and Outer Iris Circle Localization

To verify the scalability and robustness of the proposed method, the images used in this section do not intersect with the images used in Section 4.1, which belongs to an open-set test. For the CASIA-Iris-Thousand dataset, image data were randomly selected from the last 950 individuals. For the CASIA-Iris-Distance dataset, image data were randomly selected from the last 88 individuals. The specific composition of the dataset is shown in Table 2.

The traditional Daugman’s localization algorithm iteratively searches the center and radius of the circle in the entire iris image, which takes a very long time, even tens of seconds, so it has no practical significance. Based on the above considerations, the comparison of localization was based on the same size of searching field. Under the same premise, Daugman’s integro-differential operator was compared with the improved operator proposed in this paper. Table 3 and Table 4 show the accuracy of different algorithms in locating the inner and outer circles at a short distance and long distance, respectively.

**Figure 8:**
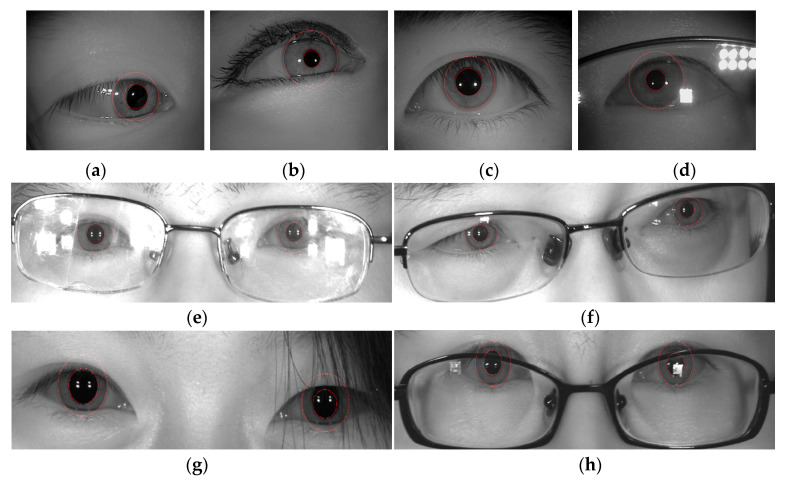
Inner and outer boundaries of iris localization by improved operators under non-cooperative environments [22]. (**a**) Off-angle iris; (**b**) iris obscured by eyelids; (**c**) iris interfered with by eyelashes; (**d**,**e**) iris with specular reflection; (**f**) off-angle iris at a long distance; (**g**) iris obstruction due to hair; (**h**) glasses obstructing the iris [22].

Table 3 and Table 4 demonstrate that the proposed operator in this study had greater accuracy than Daugman’s algorithm in images with and without glasses, which shows that the improved operator is effective and improves the accuracy of iris precise localization. Compared with Daugman’s operator, the modified operator improved the localization accuracy by 2.74% for short-distance images without glasses and by 4.06% for images with glasses. Because the compensation factors were calculated in each iteration, the localization time was a little longer. For the long-distance images, the modified operator located the iris more accurately than at a short distance, and it was much more accurate than Daugman’s operator. Especially for the images with glasses, the localization accuracy reached 84%, which is much better than Daugman’s algorithm. The success rate of localization using Daugman’s method is rather low, with only 14 irises successfully located, while no two eyes were successfully located simultaneously. It does not make sense to consider run time as a result. The experimental results show that the modified operator proposed in this paper improves the robustness of iris localization, especially under some non-cooperative environments, as shown in the Figure 8.

## 5. Conclusions

We propose a novel detection model and a modified integro-differential operator for accurate and robust iris localization in non-cooperative environments. We first used MobileNetV2 as the backbone network in YOLO v4 for feature extraction. The modified YOLO v4 model is only 5.8 M in size, which is significantly smaller than the traditional YOLO v4-tiny model. Then, a modified integro-differential operator was used to precisely locate the inner and outer boundaries. Extensive experiments on multiple datasets demonstrated the effectiveness and robustness of our method for iris localization in non-cooperative environments.

Limited by conditions, we did not find an image dataset of eye diseases. Iris localization for ocular diseases has a low success rate. This will be achieved in future research work. Furthermore, we are interested in investigating how to accurately localize the iris in video.

## Figures and Tables

**Table 1:** mAP of two detection networks under different IoU.

IoU	0.5	0.6	0.7	0.8
mAP of YOLO v4-tiny (%)	98.66	94.80	86.31	60.44
mAP of the proposed method (%)	99.83	98.49	90.57	41.50

**Table 2:** Dataset Composition.

Dataset	Number of Images without Glasses	Number of Images with Glasses
CASIA-Iris-Thousand	4000	500
CASIA-Iris-Distance	500	100

**Table 3:** Comparison results of iris localization at a short distance.

Method	Images without Glasses		Images with Glasses	
	Location Accuracy	Time Cost (s)	Location Accuracy	Time Cost (s)
Daugman’s operator	94.98%	0.215	89.85%	0.216
Proposed method	97.72%	0.227	93.91%	0.196

**Table 4:** Comparison results of iris localization at a long distance.

Method	Images without Glasses		Images with Glasses	
	Location Accuracy	Time Cost (s)	Location Accuracy	Time Cost (s)
Daugman’s operator	78.46%	2.162	7%	N/A
Proposed method	98.32%	2.213	84%	2.248

## Data Availability

Publicly available datasets were analyzed in this study. The data can be found here: http://biometrics.idealtest.org/findTotalDbByMode.do?mode=Iris#/ (accessed on 30 November 2022).

## Abbreviations

The following abbreviations are used in this manuscript:

YOLO	you only look once
AP	average precision
mAP	mean AP
*TP*	true positives
*FP*	false positive
*FN*	false negative
IoU	intersection over union
CASIA	Chinese Academy of Sciences Institute of Automation
CNN	convolutional neural network
R-CNN	region-based CNN
SPP	spatial pyramid pool
PAN	path aggregation network
